# Drop Weight Impact Test on Prepacked Aggregate Fibrous Concrete—An Experimental Study

**DOI:** 10.3390/ma15093096

**Published:** 2022-04-25

**Authors:** Gunasekaran Murali, Sallal Rashid Abid, Mugahed Amran, Nikolai Ivanovich Vatin, Roman Fediuk

**Affiliations:** 1 Peter the Great St. Petersburg Polytechnic University, 195251 St. Petersburg, Russia; vatin@mail.ru (N.I.V.); fedyuk.rs@dvfu.ru (R.F.); 2 Civil Engineering Department, University of Wasit, Kut 52001, Iraq; sallal@uowasit.edu.iq; 3 Department of Civil Engineering, College of Engineering, Prince Sattam Bin Abdulaziz University, Alkharj 16273, Saudi Arabia; 4 Department of Civil Engineering, Faculty of Engineering and IT, Amran University, Amran 9677, Yemen; 5 Polytechnic Institute, Far Eastern Federal University, 690922 Vladivostok, Russia

**Keywords:** impact load, notched specimen, bedding, result dispersion, fibers, grout

## Abstract

In recent years, prepacked aggregate fibrous concrete (PAFC) is a new composite that has earned immense popularity and attracted researchers globally. The preparation procedure consists of two steps: the coarse aggregate is initially piled into a mold to create a natural skeleton and then filled with flowable grout. In this instance, the skeleton was completely filled with grout and bonded into an integrated body due to cement hydration, yielding a solid concrete material. In this research, experimental tests were performed to introduce five simple alterations to the ACI 544 drop weight impact test setup, intending to decrease result dispersion. The first alteration was replacing the steel ball with a steel bar to apply a line impact instead of a single point impact. The second and third introduced line and cross notched specimens at the specimen’s top surface and the load applied through a steel plate of cross knife-like or line load types. These modifications distributed impact load over a broader area and decrease dispersion of results. The fourth and fifth were bedding with sand and coarse aggregate as an alternate to the solid base plate. One-hundred-and-eight cylindrical specimens were prepared and tested in 12 groups to evaluate the suggested alteration methods. Steel and polypropylene fibers were utilized with a dosage of 2.4% to produce PAFC. The findings indicated that the line notched specimens and sand bedding significantly decreased the coefficient of variation (COV) of the test results suggesting some alterations. Using a cross-line notched specimen and line of impact with coarse bedding also effectively reduced COV for all mixtures.

## 1. Introduction

In recent times, the need for social infrastructural facilities with adequate impact resistance structures has grown as damage to structures caused by impact events such as vehicle accidents and rockfall have increased. Several studies looked into the behaviour of concrete structures commonly found in infrastructure when subjected to impact loads. Under high-rate impact loading, concrete exhibits a complex behaviour that differs significantly from that observed under static loading. Consequently, impact resistance of buildings using recommendations based on general dynamic or static loading conditions or existing design standards was not suitable for evaluation. The most common design standards referred to, such as UFC 340-02 [1], ACI 349-13 [2], and ACI 544 [3], suit specific structures such as defence and nuclear plant facilities. However, the major problem of these structures is high-velocity impact loads such as explosions and aircraft hits. This indicated a reduced implementation of these standards on social infrastructural facilities, including buildings and bridges, which tend to be subjected to low-velocity impact loads like explosions and vehicle accidents than high-speed impact loads. When low-velocity impact loads are applied to structures, global reactions including rotations and displacement are the primary measures for failure of the structures. In contrast, the phenomenon of high-velocity impacts creates a structural failure at the local damage level. The global behaviour of concrete members subjected to low-speed impact loads cannot be evaluated or regulated by any single approach at this time.

Earlier investigations focused on the drop weight impact test performance for a different type of fibrous concrete. Rai and Singh [4] investigated the impact resistance of fibrous concrete using the ACI drop weight impact test. Fibrous concrete was prepared with a hybrid combination of polypropylene and steel fibers. Results showed that the impact strength of concrete comprising hybridized steel-polypropylene fibers was superior to the mono fibrous. Abid et al. [5] examined the impact performance of self-compacting fiber reinforced concrete (FRC) using the ACI 544-2R repeated blows test. Micro steel fibers mixed in three different volumes of 0.5, 0.75, and 1.0 percent were employed. The testing setup was altered to simulate different drop weights and free fall heights for multiple drop impacts. The three free fall heights adopted were 450, 575, and 700 mm, while the drop mass weight adopted was 4.5, 6.0, and 7.5 kg. Findings indicated that the impact resistance of self-compacting fiber reinforced concrete greatly improved and ranged from 150 to 860 percent than for the reference specimen. The specimens tested under conditions of 450 mm free fall height and 4.5 kg weight exhibited the most excellent resistance to impact among the five loading scenarios. Alwesabi et al. [6] investigated a low-velocity (2.8 m/s) drop hammer impact on FRC and rubber-based FRC prisms. The prism was of 100 × 100 × 500 mm size with 0%, 0.1%, 0.175, 0.25%, 1% dosages of polypropylene fiber and 0%, 0.75%, 0.825%, 0.9%, and 1.0% of micro steel fibers and 20% substitution of fine aggregate with crumb rubber. Results revealed that the prism comprising 0.1% polypropylene fiber + 0.9% steel fiber absorbed impact energy ten times higher and that impact ductile index was two times higher than that of the non-fibrous reference prism. Evolution in the technology of materials and unification increased the advent of novel forms of composite materials known as prepacked aggregate fibrous composites (PAFC), with the possibility of using them in varied engineering fields.

PAFC is referred to under different names, including two-stage fibrous concrete, colcrete, and grouted aggregate fibrous concrete. The production of PAFC differs from the production of traditional fibrous concrete in several ways. PAFC is developed by first placing coarse aggregate and fibers in an empty mold to form a skeleton. The cementitious grout is used to fill in the voids between the skeletons. PAFC has been used in various structures, including bridge foundations, caissons, bridge pier beam, and nuclear shields [7,8,9,10,11,12].

Through applying the PAFC concept, Manohar et al. [13] researched fenders made with composite steel for use in bridge pier shields subjected to vessel impact. A corrugated steel plate of 1 and 2 mm thicknesses was used in the middle portion of the fender, with the inner and outer panels of the fender being made of PAFC. Findings showed that the outer panel sustained damage at a localized level in the form of a hemispherical crater due to multiple impacts. This occurrence implied that corrugated steel plates with a thickness of 2 mm had a superior capacity of energy engrossment than 1 mm steel plates. Impact resistance from low-velocity impacts was excellent when combined with the action of corrugated steel plate and PAFC fenders. Abirami et al. [14] researched a three-layered PAFC containing 5D hooked end fiber with varying layers of glass fiber mesh and different diameters were inserted between the three layers of PAFC. The findings confirmed that the impact energy absorption properties of the larger diameter glass fiber mesh were excellent. Additionally, adding more layers of glass fiber mesh between the top and middle layers and fewer layers between the middle and bottom layers reduced impact energy absorption. The glass fiber mesh acted as an obstruction in concrete during crack growth, resulting in the postponement of the structure’s failure. However, the material’s impact resistance performance subjected to drop weight needs to be evaluated more extensively.

## 2. ACI 544 Drop Weight Impact Test

**Figure 1:**
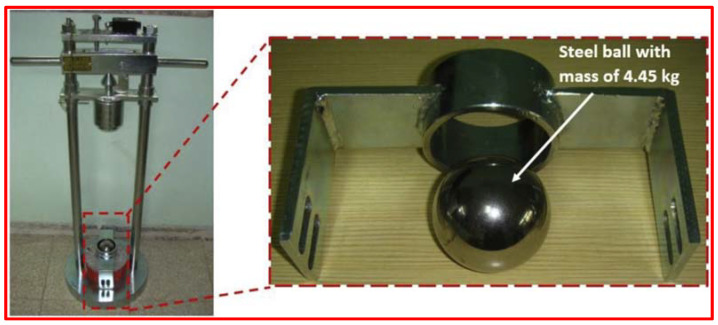
ACI Committee drop weight test (Adapted from [15]).

A different testing method is described in ACI 544-2R, but drop weight impact standardized test is believed to be the simplest. Only qualitative measurements of the FRC impact strength can be recorded and it is easy to perform this test. This test determines only the impact number without other measurements such as strain deformation and load. It can compare different types of concrete resistance to impact or the influence of mixture parameters like various fibers and dosages. Applying the ACI 544 drop weight test is very simple and is conducted by manual impact of a 152 mm diameter and 63.5 mm height cylindrical specimens. A 4.45 kg mass falls freely repeatedly from a height of 457 mm until failure occurs. The specimen is placed on a steel disc and holds a particular positioning lug and a 63.5 mm diameter steel ball on a central top, as illustrated in Figure 1 [15].

The steel ball distributes load, and petroleum jelly or grease is used under the test specimen. To avoid any lateral movement of the test specimen during the test pieces of elastomer are utilized to fill gaps between the side lugs and the specimen. The impact number is noted when the first crack becomes visible, which is the impact number needed to cause cracking. Then, testing is continued until failure occurs, which is then defined as the impact number at which the crack opens more broadly and touches the side lugs. Several researches were carried out to assess the impact behaviour of different FRC [16,17,18,19,20,21,22,23,24,25,26] and a statistical was applied to determine the number of specimens necessary to attain an error less than 10% [27,28,29,30,31,32]. However, the research outcome indicated a significant scattering of results, as shown in Table 1.

## 3. Alterations Suggested to the ACI 544 Test

Badr and Asour [33] identified several factors that contribute to a significant scattering of results for the ACI 544 test and classified them as follows:

− Breaking of the specimens is permitted in any direction and at any location. Additionally, cracking is observed visually for evaluation, which adds to the subjectivity of the test results.
− As impact applied to the specimen at a single point, thus increasing the likelihood of incorrect results in the future. The point of impact might be a softer cement matrix or a harder coarse aggregate region, depending on the circumstances.
− When it comes to specimen preparation, there is no recommended standard procedure. Therefore, specimens may have smooth mold-faced surfaces.
− Specimen failure is described by the cracks propagated to the bottom of the specimen and the apparatus lug, respectively. In some cases, even if failure occurs with an excessive crack width, the observation of failure can result in repeated impacts on the specimen.
− There is no standard indicating whether a failure pattern should be accepted or rejected, causing test results being scattered.

Despite the ACI falling mass impact test’s many advantages, scattering results are the main drawback and need to be reduced. In this research, five modifications are proposed and examined to decrease test result scattering. The first modification introduces a line of impact steel bar placed horizontally on the specimen. The second, uses a line load distributor plate to introduce a line notched specimen in the system. The third specimen is a cross-notched specimen with a cross-knife-like steel plate attached to it. The fourth and fifth alterations are sand and coarse aggregate bedding, which allow the specimens to rest on it and absorb a tremendous amount of impact energy than the previous three alterations.

### 3.1. Steel Bar, Notched Specimen, and Load Transfer Plate

**Figure 2:**
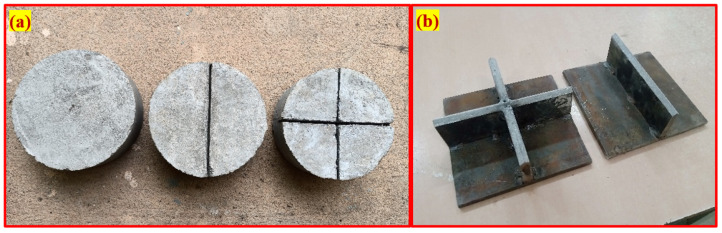
Modification details (**a**) specimens with and without notch (**b**) load transfer plates.

The first alteration introduces a steel bar of 30 mm diameter and 160 mm length being placed on the specimen’s top surface and impact load being applied on the steel bar. As a result of this alteration, cracks occur parallel to the line of impact and the specimens break into two pieces with multiple cracks in a radial direction. The second and third alterations use the topping line and cross-knife-like impact distributor plates. In the ACI testing method, a steel ball was used as a load distributor that directly transferred impact load to the specimens. In the above-mentioned two alterations, line and cross-knife-like impact distributor plates were used to replace the steel ball and the load was transferred to the notched specimens. Two different forms of the notched specimen were prepared, with a line and cross notch provided along the diameter of specimen’s top surface with a width of 3 mm and 5 mm thickness. Figure 2 illustrates the specimens with no, line, cross-notch, and load distributor plates. Crack path and failure of the notched specimens were pre-defined due to these alterations, which resulted in less result scattering.

### 3.2. Sand and Coarse Aggregate Bedding

**Figure 3:**
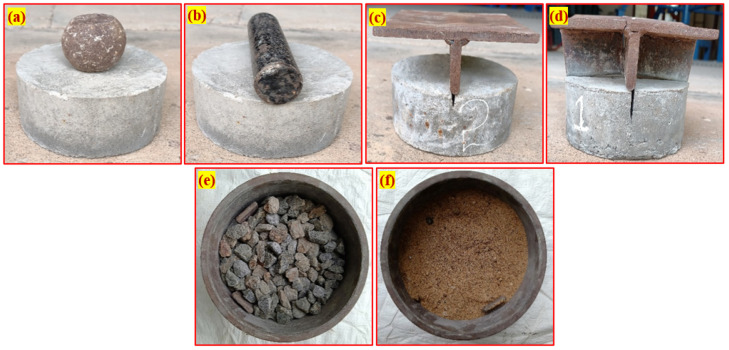
Modifications to the ACI test method: (**a**) ACI method. (**b**) Line impact load transfer by steel bar. (**c**) Line notch specimen with load transfer plate. (**d**) Cross-notch specimen with load transfer plate. (**e**) Coarse aggregate bedding. (**f**) Sand bedding.

Schrader [34] pioneered the impact resistance of concrete with the base surface of the test specimen coated with a thin grease (petroleum jelly) to decrease result scattering. In this research, uniform sand and coarse aggregate bedding were employed to study other available materials that could substitute petroleum jelly coats. The study proposed a 50 mm thick uniform sand and coarse aggregate bedding, where the specimen is embedded in 20 mm thickness. Due to its fine grading, sand was utilized where particle size was less than 2.34 mm, and crushed gravel of 20 mm size was used as coarse aggregate bedding. Simultaneously, the thickness of 50 mm bedding was recommended due to more impact energy being absorbed. In addition, the cylindrical specimen was embedded in a 20 mm thick bedding equal to one-third of the specimen’s depth. The crack that appeared on the side faces of the notched specimens could be observed visually during the test. Nevertheless, more research is needed to examine different bedding thicknesses to identify optimized thickness. Figure 3 illustrates the five different alterations proposed in this study. A cylindrical holding mold of 200 mm diameter and 150 mm height was used to locate the test specimen correctly below the impact load with the required bedding thickness. The cylindrical holding mold was about 24 mm wider than the test specimen, while the test specimens have a diameter of 152 mm. The filled sand coarse aggregate inside the holding mold is shown in Figure 3e,f. This procedure is intended to avoid the impact of specimens with holding sidewalls that can influence the crack pattern.

## 4. Research Significance

A bibliography of materials on modified testing methods for the ACI drop-weight test is not available. However, significant scattering in the ACI drop-weight test results was indicated by many researchers who used various statistical tools for analysis. Performing the modified impact test and reaching a logical conclusion to reduce scattering results is an interesting experiment and most valuable. However, only a limited amount of research was undertaken to minimize results scattering by introducing sand bedding, and there are still significant gaps in this area of study. Therefore, a modified test is necessary to reduce the scatter in the results and increase reliability. In this paper, five alterations to the ACI drop weight impact test were conducted to achieve the impact performance of PAFC. Five simple alterations to reduce scattering of results are proposed, and they involve a steel bar positioned horizontally to apply a line of impact, line, and cross-notched specimens with load distributor plates, sand and coarse aggregate bedding. The impact number associated with cracking and failure, as well as the failure mode and ductility index were thoroughly investigated.

## 5. Experimental Program

### 5.1. Raw Materials

**Figure 4:**
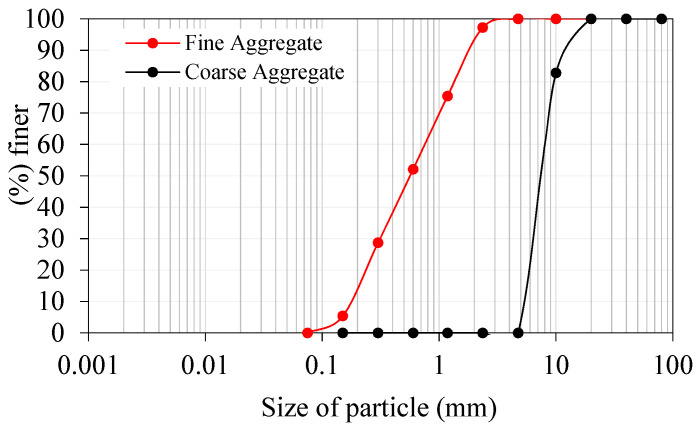
Gradiometric curve for the used aggregates.

**Figure 5:**
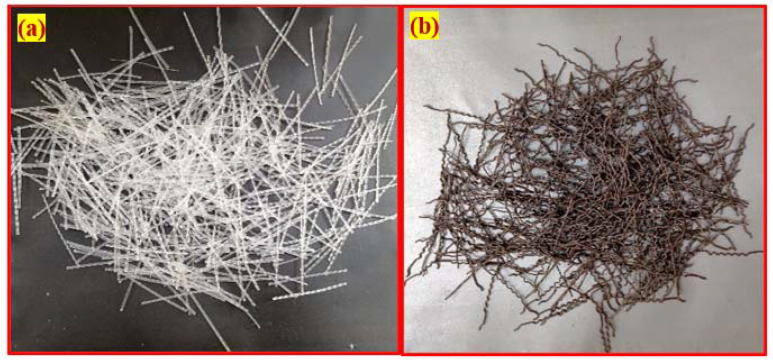
Fibers type used in this research: (**a**) Polypropylene. (**b**) Steel.

− An IS: 12269-1987 [35] compliant Dalmia Pozzolana Portland obtained from Tamil Nadu, India, a general-purpose cement was used in the studies. The cement had a specific gravity of 3.09 kg/m^2^ and a specific surface area of 318 kg/m^2^.
− When using river sand as fine aggregate in the final product, it had fineness modulus 2.41, water absorption 1.15, and density 2.65%. The sand had a granulometric curve that conformed to IS: 383-2016 [36], in line with ASTM C939/C939M-16a [37], with a particle size of not more than 2.36 mm. Because of this, a flowable grout was developed to fill up the gaps.
− Coarse aggregate consisted of crushed granite gravel with a maximum size of 12.5 mm, a water absorption of 0.59%, and a specific gravity of 2.69%. Figure 4 shows the obtained granulometric curves for the fine and coarse aggregates used in this experiment.
− A high-range water reducer (Tec Mix 640 obtained from New era construction chemicals, Tamil Nadu, India) was utilized to make flowable grout. For non-fibrous and fibrous specimens, different dosages (0.4 to 0.5 percent by cement weight) were used to make flowable grout.
− A new geometrically formed hybrid hooked end-crimped steel fiber (SF) of 50 mm length and 1.0 mm diameter with a tensile strength of 1200 MPa was employed. The tensile strength of polypropylene PF was 500 MPa and the fiber was 45 mm long and 0.8 mm in diameter. The appearance of SF and PF used in this research are shown in Figure 5.

### 5.2. Details of Mixing Composition

Thirty-six mixtures were employed in this investigation and were into divided into twelve groups based on the modifications proposed. A series of trials was conducted to select optimum flowable grout based on efflux time from the cone test, which met all compressive strength requirements. Optimized grout was attained based on the efflux time which ranged from 35 to 40 s as per ASTM C939 [37]. The optimal proportion of cement to sand and w/c (water to cement ratio) was 1.0 and 0.42. A high-range water reducer was added to water to enhance the flowability of grout, whose dosage was 0.4 percent for non-fibrous specimens and 0.5 percent for fibrous specimens. The composition of the twelve mixtures is shown in Table 2. The first mixture from group one was considered as reference concrete prepared without fiber, no notch, and no bedding and designated as RC-NN-NB. The second and third mixtures were prepared with PF and SF, respectively. These two mixtures comprised no notch and no bedding and were designated RC-PF-NB and SF-NN-NB. From the above three mixtures, the first letter “RC” represents the type of concrete, the second letter “NN” the type of notch, and the third letter NB the type of bedding. The second group mixtures id was based on group one, except the middle letter ST, which meant the steel bar. The middle letters of all twelve groups, NN, ST, LN, and CN, represent the no notch, steel bar, line notch, and cross-line notch, respectively. The first and third represent the type of concrete and type of bedding, respectively. Table 2 reveals the mixing combination of all twelve group mixtures.

### 5.3. Specimen Preparation Procedure

**Figure 6:**
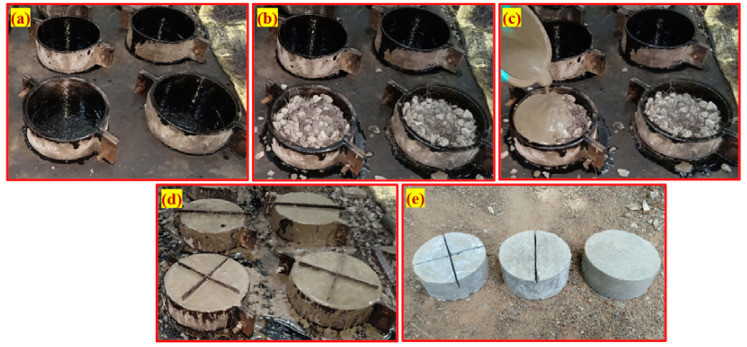
Fabrication technique: (**a**) empty mold, (**b**) filled fibers and aggregates into mold, (**c**) pouring of grout, (**d**) inserted notch plates into the specimens, and (**e**) specimens appearance after demolding.

In total, 108 cylindrical specimens were prepared with three for each mixture. The impact strength of the PAFC was measured using a 152 mm diameter and 64 mm height cylindrical specimen. As illustrated in Figure 6, the stage-by-stage process of PAFC casting consists of three critical procedures. First, a lubricant was applied to all of the cylindrical mold’s interior side faces, as shown in Figure 6a, before it was filled. Second, a natural skeleton was created by combining pre-mixed aggregates and fibers, as shown in Figure 6b. As a final step, cement grout was applied and allowed to fill the spaces under gravity. The grout was slightly compacted to ensure that all voids were filled, as shown in Figure 6c. This compaction process eradicated voids and honeycombing. Figure 6d shows the appearance of specimens after grouting with the insertion of notch plates. All specimens were demolded after 24 h and their appearances were arranged in a sequence as shown in Figure 6e. Water immersion curing for 28 days was used to cure the specimens before testing.

### 5.4. Drop Weight Impact Test Setup with Modifications

**Figure 7:**
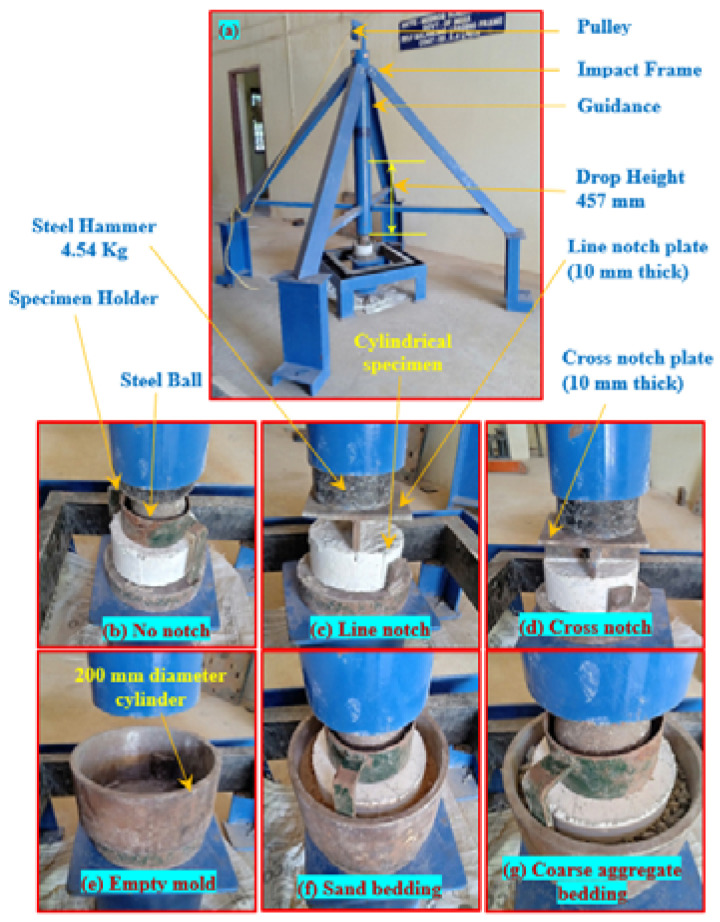
Drop weight test setup and arrangements.

An altered drop-weight impact test was conducted to evaluate impact strength of the PAFC specimens based on alterations suggested by ACI Committee 544 [3]. Figure 7 displays the modified drop-weight impact testing device used in this research. In this altered test, a mass of 4.45 kg of steel hammer freed from 457 mm height was allowed to fall on the same spot on the specimen’s top surface, as shown in Figure 7a. During testing, the specimen’s movement horizontally was restricted by four positioning legs as shown in Figure 7b–d. Impact numbers needed to produce cracking (L1) and failure (L2) of the specimen was noted by manual inspection. Failure was defined by the crack reaching the specimen’s bottom and breaking into two pieces. The empty holding cylindrical mold, sand, and coarse aggregate bedding arrangements are seen in Figure 7e–g.

## 6. Discussion of Results

### 6.1. Compressive Strength

**Figure 8:**
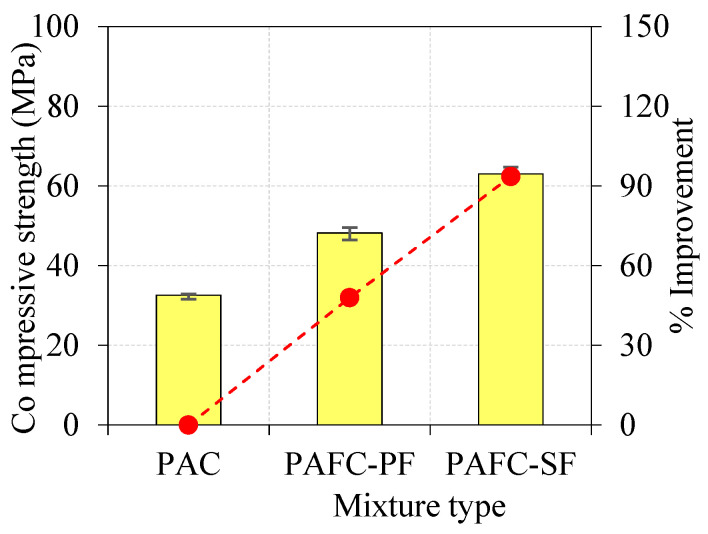
Compressive strength of concrete.

Figure 8 shows the compressive strength of both non-fibrous and fibrous concrete, attained from three 100 mm cubes per mixture at 28 days according to IS: 516 [38]. The non-fibrous specimen (PAC) had a compressive strength of 32.57 MPa, whereas the fibrous specimens comprising PF and SF had 48.20 and 63.04 MPa, respectively. It is clear from Figure 8 that the compressive strength of PAFC comprised PF and SF exhibited a 47.9% and 93.5% improvement, respectively, compared with PAC.

This was due to the three-dimensional orientation of SF and PF, which can be dispersed uniformly throughout the cross section, resulting in inhibiting macroscale crack formation. This ensured that stress was distributed uniformly while altering the crack path lead to fiber bridging action that prevented crack advancement from progressing further. The contribution of SF in increasing compressive strength was more remarkable than in PF. This finding is aligned with the earlier studies reported in [24,39,40]. This phenomenon was due to the higher tensile strength of SF leading to a higher load to maintain cracking progress around the SF. However, the addition of PF also improved compressive strength by 47.9%, which was comparatively less than that of SF. This improvement was due to the low tensile strength of PF with lesser crack bridging ability resulting in smaller energy being needed for fiber debonding and pull out [41,42,43].

### 6.2. Repeated Impact Test

**Figure 9:**
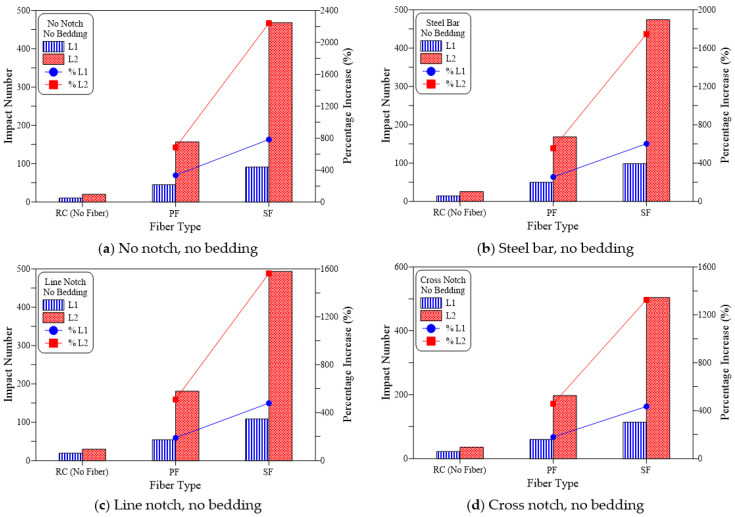
Fiber type effect on cracking and failure impact numbers of no bedding specimens (**a**) No notch, no bedding, (**b**) Steel bar, no bedding, (**c**) Line notch, no bedding (**d**) Cross notch, no bedding.

**Figure 10:**
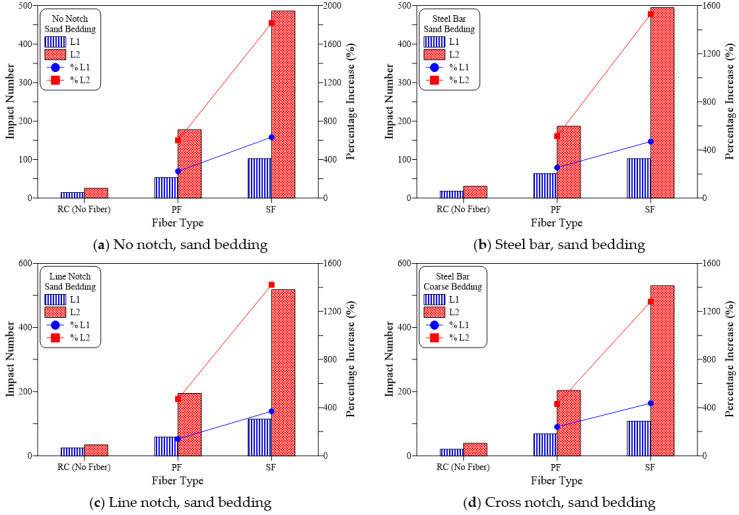
Fiber type effect on cracking and failure impact numbers of sand bedding specimens. (**a**) No notch, sand bedding, (**b**) Steel bar, sand bedding (**c**) Line notch, sand bedding (**d**) Cross notch, sand bedding.

**Figure 11:**
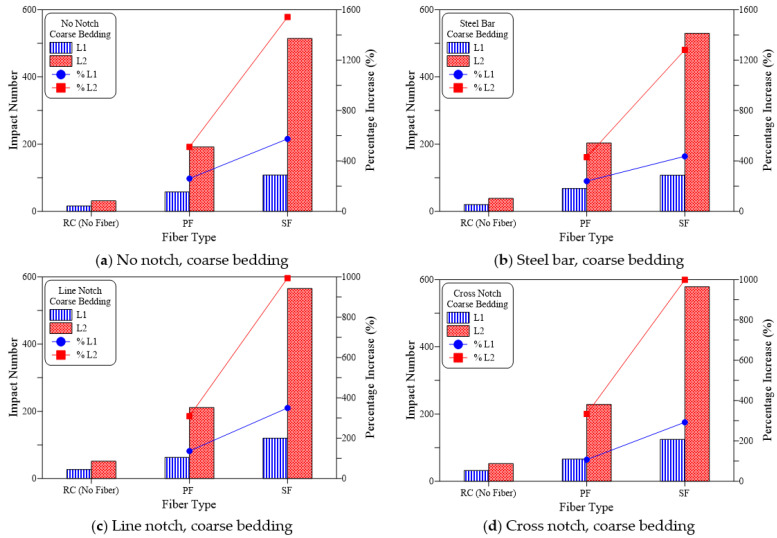
Fiber type effect on cracking and failure impact numbers of coarse bedding specimens. (**a**) No notch, coarse bedding, (**b**) Steel bar, coarse bedding, (**c**) Line notch, coarse bedding, (**d**) Cross notch, coarse bedding.

The findings recorded from the repeated impact tests are summarized in Table 3 and shown in Figure 9, Figure 10 and Figure 11 for all groups of specimens. The results are presented in terms of cracking impact number (L1) and failure impact number (L2). As shown in Table 3, the mean of three disk specimens was considered in the figures. Figure 9, Figure 10 and Figure 11 reveal the effect of the inclusion of polypropylene fiber (PF) and steel fiber (SF) on impact resistance in terms of L1 and L2, in addition to the percentage of increase compared to the corresponding plain reference specimens (RC).

#### 6.2.1. Effect of Fiber Type

Figure 9 presents the impact test results of the no-bedding groups and the four loading cases. It is obvious in the figure that PF resulted in a significant increase in both cracking and failure numbers. Whereas in this case, the percentage increase in L1 over the plain specimens (RC) was in a range from 180% to 336% for the four loading cases as shown in Figure 9a–d, which meant that the recorded L1 for the PF-reinforced specimens of the groups was approximately 2.8 to 4.4 times that of the RC specimens. Improvement in the impact failure resistance of the tested specimens was much more obvious than at the cracking stage, where L2 of the PF-reinforced specimens was increased by 458 to 685%, which meant that PF effectiveness was more than twice at failure than at cracking. Similar results were also obtained for the sand bedding groups (Figure 10) and coarse bedding groups (Figure 11). As shown in Figure 10, L1 increased by 127% to 279% compared to the RC specimens, while L2 increased by 468% to 601%. Thus, the percentage increase in L2 was more than 2.1 times that of L1. Similarly, for the coarse bedding specimens (Figure 11), percentage improvement in cracking resistance was 107% to 260%, while that of failure resistance was approximately 309% to 512%. Therefore, L2 increased by approximately 2.0 to 2.9 times than that of L1.

Improvement in impact resistance was attributed to the micro and macro size of fibers used in concrete. This type of micro-structural scale of fiber would reduce the initiation of cracks under the effect of stresses from external loads through a bridging action [44]. The bridging activity of fibers restricted the widening and propagation of cracks and delayed their opening to the outer surfaces, which in turn increased the load absorption capacity till surface cracking [45,46]. Within the first few impacts, the material started to fracture at the microstructural level, which was revealed as a multi-cracking response under gradually increasing tensile stresses. These internal cracks were rapidly connected and propagated to the surface of the reference (RC) specimens after approximately 10 to 32 impacts. On the other hand, the presence of PF fibers across internal cracks postponed propagation of these cracks by absorbing significant tensile stresses before fiber elongation allowing for partial opening of cracks, which delayed surface cracking reflecting a higher impact energy absorption capacity, where L1 of PF specimens ranged from approximately 45 to 68 impacts.

As the impact continued after cracking, the surface cracks of the plain RC specimens became wider under each impact that was transferred into concentrated tensile stresses, leading to failure fracture after just a few blows after surface cracking. All RC specimens failed after approximately 20 to 53 impact blows. On the other hand, the PF-reinforced specimens exhibited a much higher ability to absorb impact energy after surface cracking till failure compared to the RC specimens. The superior impact response of the PF specimens after cracking was due to the full use of fiber potential to absorb high tensile stresses across cracks before rupture or bond failure. The main action of fibers starts after the fibers were stressed by tensile forces at the opposite fiber ends anchored inside the opposite faces of the widening cracks. Therefore, as impact blows increased after cracking, the function of fibers as bridging units was more pronounced, reaching the full functionality before failure, which explains the much higher percentage increase in L2 compared L1 over their corresponding records of plain specimens. The distinguished impact response of PF reinforced specimens was also reported by previous researches [24,39], while others showed that PF fibers significantly improved flexural performance, toughness and ductility of concrete [47].

Figure 9, Figure 10 and Figure 11 shows clearly that L1 and L2 records of steel fiber (SF) reinforced specimens were much higher than their corresponding RC specimens and noticeably superior to the PF specimens. For the no-bedding groups, the SF specimens showed an increase between 435% to 784% for L1 and 1326% to 2242% for L2. Similarly, for the sand-bedding groups, L1 and L2 increased by 333% to 633% and 1354% to 1820%, respectively, while they increased by 293% to 575% and 994% to 1543%, respectively, for the coarse-bedding groups. Comparing the impact test results of SF specimens with their corresponding PF specimens for the 12 groups of specimens, the SF-reinforced specimens exhibited L1 records that were approximately 1.6 to 2.0 times those of PF-reinforced specimens. Similarly, L2 values of SF specimens was approximately 2.0 to 3.0 times those of PF specimens.

The superiority of SF specimens over RC specimens was due to the matrix reinforcement action of fibers, which boosted the specimens impact energy capacity and lead to improved material behaviour under loads and higher impact strength. On the other hand, the noticeably higher impact performance of SF specimens over the PF specimens was due to higher tensile strength and bond strength of the steel fibers compared to the PF fibers, where tensile strength of the used SF fibers (1150 MPa) was more than twice that of PF fibers (500 MPa). Previous studies support the findings of these results, where SF showed extraordinary reinforcing activity leading to significantly improved toughness, tensile strength, and impact resistance [46,47].

#### 6.2.2. Impact Ductility

The ability of flexural members like beams and slabs to withstand plastic deformation is defined as flexural ductility, and the ductility index is calculated by dividing the deformation (deflection) at failure or at a defined point within the post-peak region to that at the yielding point of steel tension [48,49]. In this definition, the relation between load and deformation is assumed to be elastic before yielding and plastic beyond which. If such a definition can be used to describe the ability of the disc specimens to absorb impact energy within plastic energy, then the cracking number L1 can be assumed to be the end point of elastic behaviour and the starting point of plastic behaviour. Therefore, an impact ductility index (ID) can be simplified as the ratio of the failure impact number (L2) to the cracking impact number (L1). This definition was adopted in many earlier studies to distinguish between the effects of different fiber materials or contents on the impact response under repeated impact loads [50,51,52].

**Figure 12:**
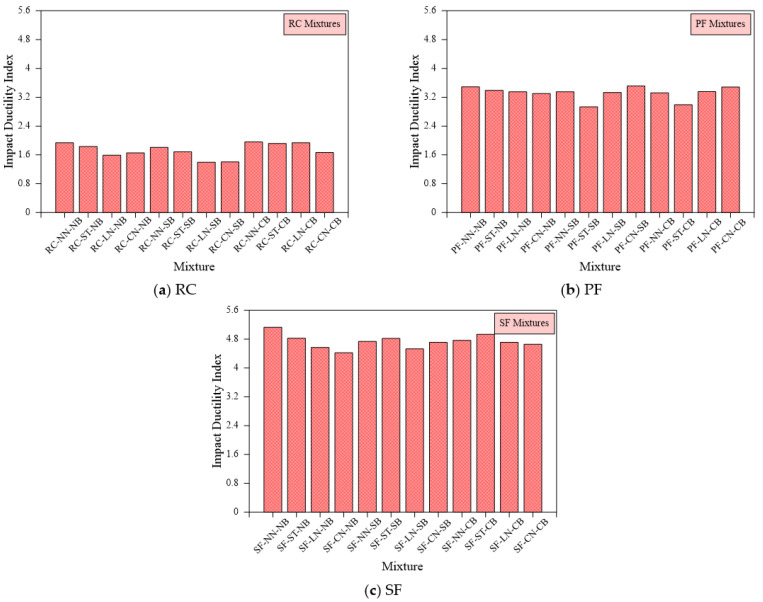
Impact ductility index of all mixtures (**a**) RC, (**b**) PF (**c**) SF.

Figure 12 shows the impact ductility index (ID) for the 12 bedding and loading cases for the RC, PF, and SF specimens. Figure 12a reveals that ID values for the RC specimens are comparable for all cases which range from 1.40 to 1.96. On the other hand, Figure 12b reveals the effect of PF in improving impact ductility where ID values of the PF reinforced specimens range from 2.93 to 3.51. Comparing these values with ID values of the plain RC specimens, it is seen that the ductility indices of PF specimens were 1.56 to 2.50 times those of RC specimens. This improvement is attributed to better post cracking performance of the PF specimens due to the bridging action of PF fibers which delayed failure and increased differences between L2 and L1 records. Figure 12c shows that the SF-reinforced disk specimens exhibited comparable ID values in a range of 4.42 and 5.13. The calculated ID values for the FS specimens reveal the superior impact ductility of SF disks compared to plain and PF disks. The ductility of SF specimens is obviously 2.43 to 3.36 times that of RC specimens and 1.34 to 1.65 times that of PF specimens. The higher ductility of SF is attributed to the same reason ascribed in the previous section, which is the higher tensile strength and better configuration of steel fibers compared to polypropylene fibers, which in turn increased crack arresting potential after the first crack leading to much higher L2 records compared to corresponding L1 records.

## 7. Analysis of the Recommended Changes to ACI 544-2R Repeated Impact Test

### 7.1. Effect of Loading Type on Impact Results

**Figure 13:**
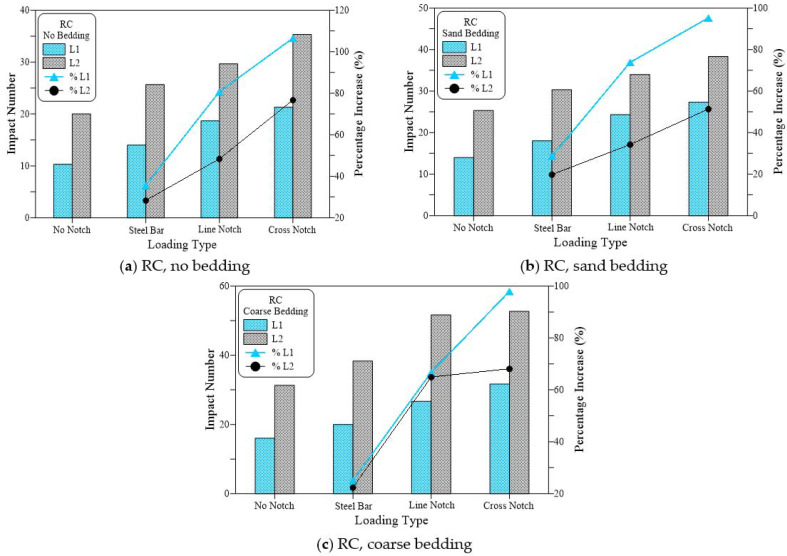
Loading type effect on cracking and failure impact numbers of RC specimens. (**a**) RC, no bedding, (**b**) RC, sand bedding (**c**) RC, coarse bedding.

**Figure 14:**
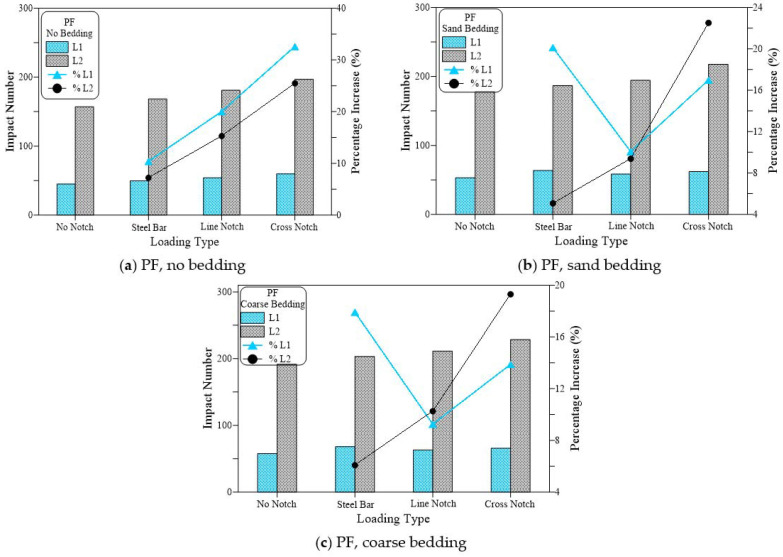
Loading type effect on cracking and failure impact numbers of PF specimens. (**a**) PF, no bedding, (**b**) PF, sand bedding (**c**) PF, coarse bedding.

**Figure 15:**
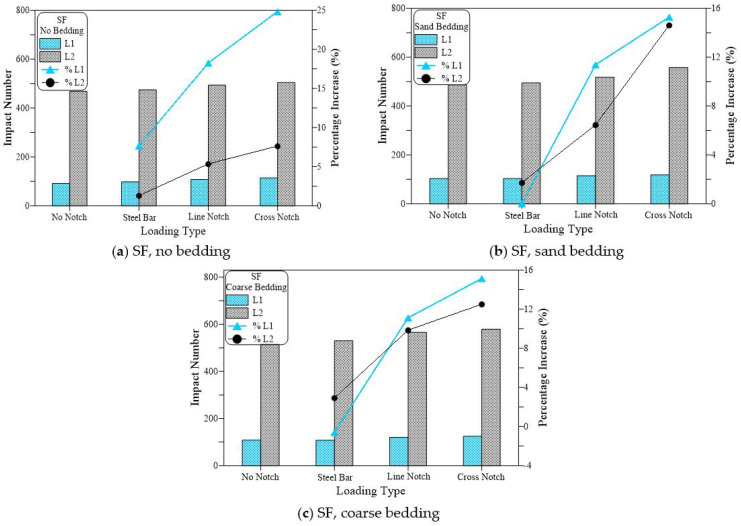
Loading type effect on cracking and failure impact numbers of SF specimens. (**a**) SF, no bedding, (**b**) SF, sand bedding (**c**) SF, coarse bedding.

Figure 13, Figure 14 and Figure 15 compare the cracking (L1) and failure (L2) impact results of the three suggested loading types (steel bar, line notch and cross notch) with the standard steel ball configuration (no notch). In each of these figures, the results are compared for the three bedding cases of the standard no bedding, sand bedding, and coarse bedding.

Figure 13 presents the results of the plain (no fiber) specimens (RC). Figure 13a shows that for the case of no bedding, testing the specimens under the three testing techniques increased the retained impact numbers of the disc specimens, where the impact numbers (L1 and L2) of the steel beam case were higher than those of the standard no notch case, while using line notch and line knife plate resulted in higher impact numbers. However, the highest retained impact numbers of all cases were recorded for the case of cross notch and cross loading plate. The retained L1 values were 10, 14, 19, and 21 for the cases of no notch, steel beam, line notch and cross notch, respectively. Therefore, the alternative loading techniques of steel beam, line notch, and cross notch increased L1 by 35%, 81%, and 106%, respectively, compared to the standard loading case. Similarly, L2 values of the same loading cases were 20, 26, 30 and 35 recording percentage increases over the standard case by 28%, 48%, and 77%, respectively. Similar trend and sequence of results were obtained for the similar groups of loading cases tested with sand bedding (Figure 13b) and coarse bedding (Figure 13c). Whereas shown in Figure 13b, in the case of sand bedding, L1 increased by 29%, 74%, and 95% for the cases of steel beam, line notch and cross notch, respectively, compared to the no notch case, while L2 exhibited percentage increases of 20%, 34%, and 51%, respectively. The group of coarse bedding also supports these results with percentage increases of 25%, 67%, and 98% in L1 and 22%, 65%, and 68% in L2, respectively, as shown in Figure 13c.

Figure 14 shows a similar trend of results recorded for the polypropylene fiber-reinforced mixture (PF), while Figure 15 shows these results for the steel fiber-reinforced mixture (SF). This is similar to the trend observed in L1 and L2 for the plain mixture (Figure 13). Where the suggested loading cases of the steel beam, line notch, and cross-notch exhibited higher L1 and L2 records than the standard loading case (no notch). However, the percentages increase in L1 and L2 of the fibrous mixtures over the standard loading case were much lower than those recorded for the plain mixture. The percentages increase in L1 recorded for the PF mixtures for was 10%, 20%, and 33%, the no bedding 20%, 10%, and 17% for the sand bedding and 18%, 9%, and 14% for the coarse bedding for the steel beam, line notch and cross notch, respectively. For the same sequences of bedding types and loading types, the percentages increase in L2 was 7%, 15% and 25%, 5%, 9% and 23%, and 6%, 10%, and 19%, respectively, as shown in Figure 14.

This means that the percentage increase in both L1 and L2 was in the range of 5 to 33% for the PF specimens if the suggested loading cases were used instead of the standard steel ball (no notch). Figure 15 shows that the SF-reinforced mixtures also exhibited a similar trend of results. However, with a much lower percentage of increase that ranged from −1 to 25% for L1 and 1 to 15% for L2. On the other hand, the percentage increase in L1 for the case of the plain mixture (Figure 13) ranged from 25 to 106%, while that of L2 ranged from 20 to 77%, which are noticeably higher than those retained for the PF and SF fibrous mixtures. The obtained results agree well with those obtained in a previous study for the two notch cases [53], while the steel bar case was first introduced in this research. In the previous study by Ramakrishnan et al. [53], it was revealed that regardless of the bedding case, using line or cross notch loading cases increased the retained impact numbers.

### 7.2. Effect of Bedding Type on Impact Results

**Figure 16:**
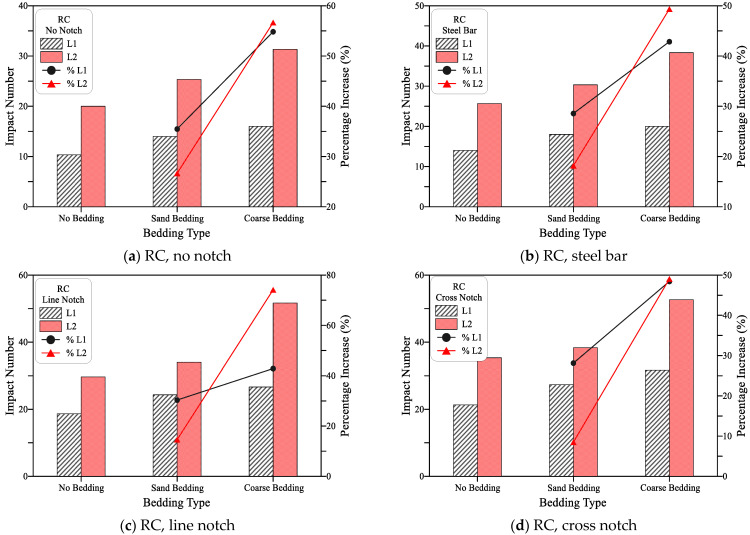
Bedding type effect on cracking and failure impact numbers of RC specimens (**a**) RC, no notch, (**b**) RC, steel bar, (**c**) RC, line notch (**d**) RC, cross notch.

**Figure 17:**
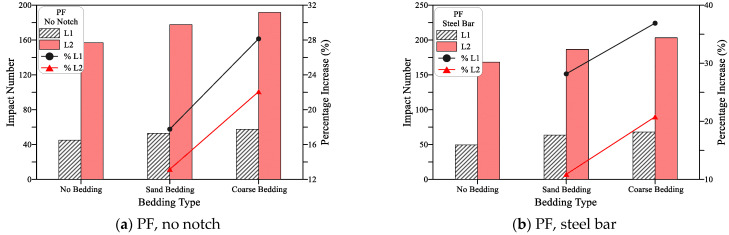

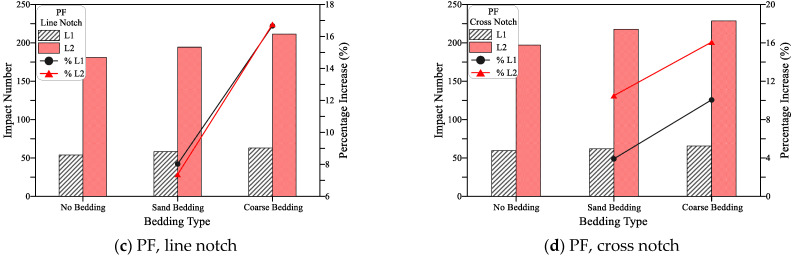
Bedding type effect on cracking and failure impact numbers of PF specimens (**a**) PF, no notch, (**b**) PF, steel bar (**c**) PF, line notch (**d**) PF, cross notch.

**Figure 18:**
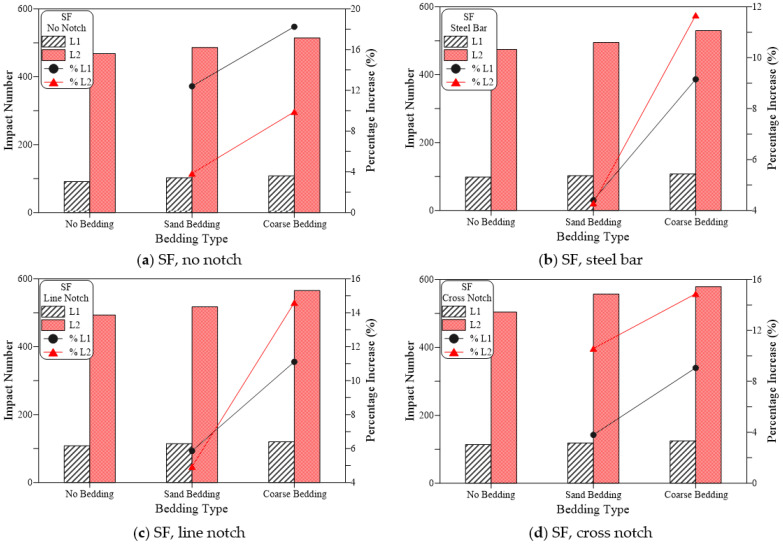
Bedding type effect on cracking and failure impact numbers of SF specimens (**a**) SF, no notch, (**b**) SF, steel bar, (**c**) SF, line notch (**d**) SF, cross notch.

Figure 16, Figure 17 and Figure 18 illustrate the effect of bedding type on the recorded impact numbers for the plain, PF, and SF mixtures, respectively, in which this effect is shown for the four loading cases. It is obvious in the figures that using bedding materials increases the obtained impact numbers and this increase is higher for coarse bedding than for sand bedding, regardless of the case of loading and mixture type. Figure 16 shows that for RC mixtures, the use of sand bedding increased L1 from 28 to 35% and L2 by 8 to 28% for all loading cases. On the other hand, the use of coarse bedding increased L1 by 43 to 55% and L2 by 49 to 74% for the four loading cases. For the PF mixture, Figure 17 shows that using sand bedding increased L1 and L2 by 4 to 28% and 7 to 13%, respectively, compared to the similar load cases without bedding, while the use of coarse bedding led to higher L1 records by 10 to 37% and higher L2 records by 16 to 22% compared to their corresponding loading cases without bedding. The percentage increase values in the PF mixture is noticed to be apparently lower than those gained for RC mixture. This conclusion was also recorded for the SF mixture but with much lower increase percentages as shown in Figure 18, where the use of sand bedding resulted in an increase in L1 and L2 between 4% and 12%, while the using of coarse bedding increased L1 and L2 by 9 to 18%.

The increase in the retained impact numbers due to the use of bedding materials was also reported by previous researches [28,53]. This increase is directly attributed to the relief in the absorbed impact energy by the disc specimen obtained from the partial energy absorbance of the bedding materials. The compaction of the bedding materials absorbs a part of the applied impact load leading to lower stresses in the disc specimen after each impact blow. Therefore, the degradation of concrete that leads to the cracking and failure is delayed resulting in higher retained impact numbers till cracking and failure. Ramakrishnan et al. [53] reported an increase in L1 and L2 from 45 to 350% when sand and coarse bedding materials were used. On the other hand, the lower recorded percentage increase values for PF and SF mixtures compared to the RC plain mixture is directly attributed to the presence of fibers as discussed in the previous section [54,55,56,57].

### 7.3. Effect of Loading Type and Bedding Type on the Dispersion of Impact Results

The Coefficient of Variation (COV) is a comparatively non-dimensional measure of variability and is a significant parameter in the building design industry. The principle helps to simplify statistical parameter calculations, and the outcomes are very close to those obtained directly from the original functional relationship. By finding the uncomplicated relationship between COV and statistical parameters, statistical distribution characteristics can be analyzed by COV. A higher value of COV indicates higher variability in the testing data point, while a lower COV indicates a lower variability desirable for design calculations. The effect of loading and bedding type on the COV of impact test results, is shown in Figure 15. The assessment of impact of the proposed loading and bedding types should not be based solely on L1 and L2 values. Furthermore, variation control in the test was one of the key objectives for suggesting these changes [3]. Table 3 lists the SD and COV values of L1 and L2. Consequently, the COV was adopted to compare the scattering of results between various loading and bedding types and other mixing combinations in this research.

**Figure 19:**
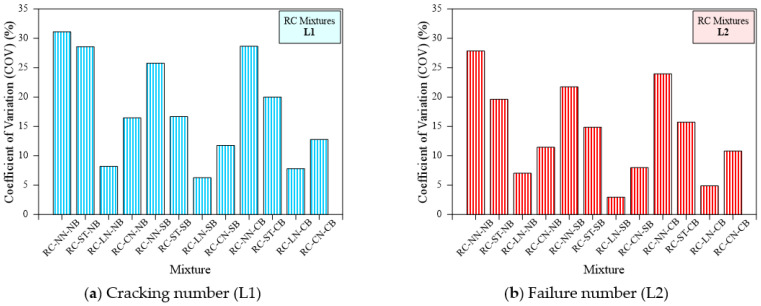
Coefficient of variation of cracking and failure impact numbers of RC specimens (**a**) Cracking number (L1), (**b**) Failure number (L2).

Figure 19 illustrates the COV for each RC type mixture for all loading and bedding types. The figure indicates a declining trend of COV in comparison to each no bedding and ACI 544 method loading type. For example, the determined COV of L1 for the RC-NN-NB, RC-ST-NB, RC-LN-NB, RC-CN-NB specimens was 31.1%, 28.6%, 8.2% and 16.5%, respectively. Likewise, COV values for L2 were 27.8%, 19.6%, 7.0%, 11.4%, respectively, as in Figure 19. It is clear from the figure and the ACI testing method that they exhibited a higher COV and this variability was reduced by altering the loading type. The lowest COV was obtained from the line notched specimens followed by the cross notched specimens and no notch specimens with a line of impact using a steel bar. A specimen exhibited the same trend with sand and coarse aggregate bedding. On the other hand, sand and coarse aggregate bedding exhibited a positive result in reducing the variability of specimens under impact loading. For example, the COV of L1 for the RC-NN-NB, RC-NN-SB and RC-NN-CB specimens was 31.1%, 25.8%, and 28.6%, respectively, while the OCV for L2 values was 27.8%, 21.7%, and 24.0%, respectively. It is clear from Figure 19 that the specimen with sand bedding displayed lower results variability followed by coarse aggregate bedding. COV values ranged from 8.2 to 31.1% for no bedding, 6.3 to 25.8% for sand bedding and 7.8 to 28.6% for coarse aggregate bedding corresponding to L1. Similarly, COV for L2 ranged from 7 to 27.8% for no bedding, 2.9% to 21.7% for sand bedding and 4.9 to 24% for coarse aggregate bedding irrespective of the loading type and the specimen. Comparing RC specimens with different loading and bedding types, a notable COV reduction was obtained from the line notched specimen with sand bedding.

**Figure 20:**
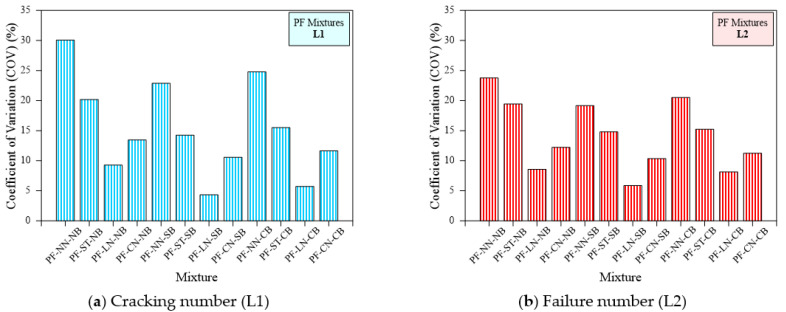
Coefficient of variation of cracking and failure impact numbers of PF specimens (**a**) Cracking number (L1), (**b**) Failure number (L2).

**Figure 21:**
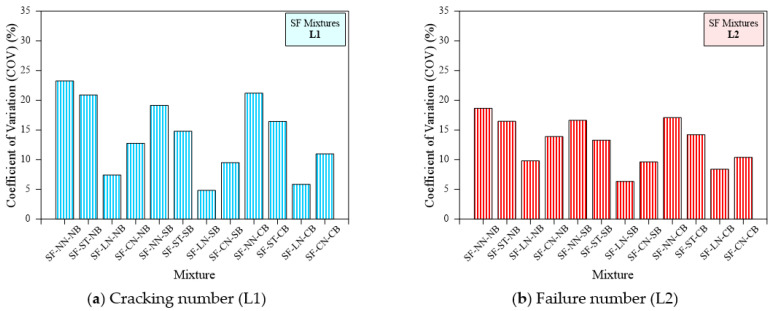
Coefficient of variation of cracking and failure impact numbers of SF specimens (**a**) Cracking number (L1), (**b**) Failure number (L2).

The same trend was observed in polypropylene fibrous specimens. Figure 20a,b illustrates the COV for each type of specimen comprising PF. For example, the COV of L1 for the PF-NN-NB, PF-ST-NB, PF-LN-NB, and PF-CN-NB specimens was 30.1%, 20.2%, 9.3%, and 13.4%, respectively, while the COV for L2 values were 23.8%, 19.4%, 8.6%, and 12.2%, respectively. A similar trend was observed for the sand and coarse aggregate bedding. Comparing the bedding type, the observed COV values for the PF-NN-NB, PF-NN-SB, and PF-NN-CB were 30.1, 22.9, and 24.8 for L1 and 23.8, 19.2, and 20.5 for L2, respectively. The use of line notch specimens with sand bedding displayed a lower COV for both the L1 and L2 of PF-based specimens. SF-based specimens also exhibited the same trend as observed in the non-fibrous and PF-based specimens, shown in Figure 21. For example, SF-NN-NB, SF-ST-NB, SF-LN-NB, and SF-CN-NB specimens displayed a COV of about 23.2%, 20.9%, 7.4%, and 12.7% for L1 and 18.6%, 16.4%, 9.8%, and 13.9% for L2, respectively. It is clear from Figure 21, that the sand bedding with line notched specimens exhibited a lower COV value. In a nutshell, the line notched specimen showed the smallest scattering, while the line notch with sand bedding was the solution to decrease the scattering of results.

### 7.4. Failure Pattern of a Specimen under Impact Loading

**Figure 22:**
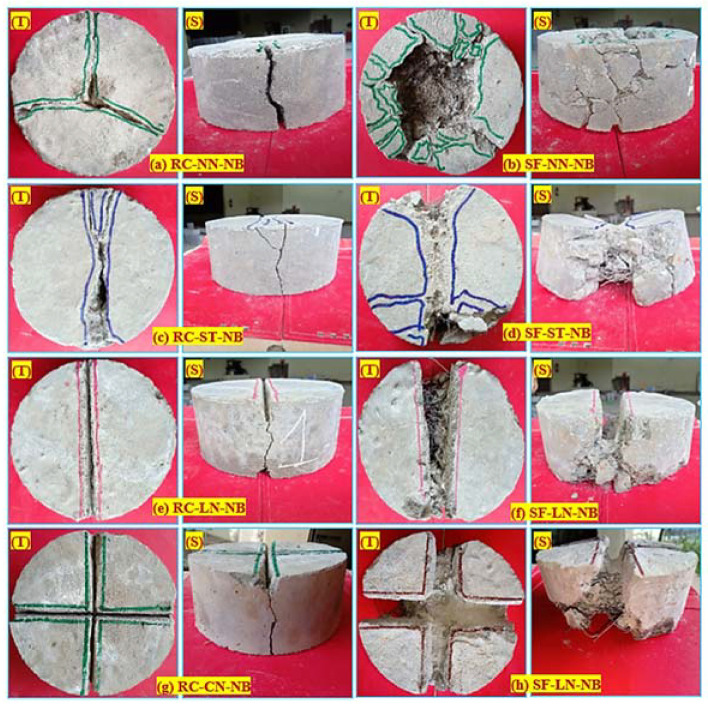
Failure pattern. (**a**) RC-NN-NB **(b**) SF-NN-NB (**c**) RC-ST-NB (**d**) SF-ST-NB (**e**) RC-LN-NB (**f**) SF-LN-NB (**g**) RC-CN-NB and (**h**) SF-LN-NB; where T─top and S─Side.

Figure 22 shows the failure of the PAFC specimen on the top and side faces under repeated impact load for the non-fibrous and fibrous specimens subjected to no bedding. The failure pattern of the sand and coarse aggregate bedding specimens was similar to the corresponding alterations proposed by the specimen. However, alterations such as steel bars, line and cross notched specimens showed a different failure pattern. As shown in Figure 22a, the RC-NN-NB showed brittle failure while the specimen’s crack path occurred randomly. On the other hand, the SF-NN-NB specimen showed ductile failure due to the presence of SF. The central semi-circular fraction zone under the load-distributor steel ball was formed, accompanied by several macro cracks that showed the specimen’s high energy absorption capacity, as demonstrated in Figure 22b. Specimens subjected to line impact (RC-SB-NB and SF-SB-NB) showed that a crack occurred parallel to the line of impact and spreads to the bottom of the specimen, leading to a breakage in two parts of the specimen, as shown in Figure 22c,d. The notched specimens (line and cross) were found to control crack paths better.

The crack spread along with the depth of the notches as the frequency of impacts increased, as seen in Figure 22e–h. The lines and cross notched specimens produced fractures on two and four sides, respectively, due to the impact of the loading notches and distributors that distributed stress along a cross-line controlling the path of fractures. This failure pattern was consistent with previous studies [28,53]. A more even line cracking was replaced for fibrous specimens in the central semi-circular fracture zone under the load-distributor steel ball. This showed the successful role of stress direction and crack path controlling the proposed line loading distributor and the notched specimens. Such control could reduce the variability of test results and help suggest more control criteria for cracking and the specimens’ failure through repeated impact testing [58,59,60,61]. For single-layer, double-level, and three-layered specimens, the same failure observation was recorded.

## 8. Conclusions

Based on the findings of the experiments and the statistical analysis, the following conclusions are reached.

− The three suggested loading cases of steel bar, line notch, and cross-notch enabled the disc specimens to absorb higher impact energy compared to the standard steel ball case, which is attributed to the better distribution of stress concentration along line and cross baths compared to the single central point in the case of the standard steel ball. For instance, for the RC mixture, the percentage of increase in L1 and L2 for the three suggested loading cases was in the ranges of 25% to 106% and 20% to 77%, respectively, compared to the corresponding L1 and L2 records of the standard steel ball case.
− The use of sand and coarse beddings led to a kind of stress relief in the disc specimens under the repeated impacts owing to the partial compaction deformation of the bedding material. This relief increased the retained cracking and failure impact numbers compared to the standard case without bedding, regardless of the surface loading mechanism. For instance, the retained L1 numbers of the RC mixture increased by 28% to 55% when sand or coarse bedding materials were used, while L2 increased by 8% to 74% compared to the standard case without bedding.
− The influence of suggested surface loading type and bedding materials on the retained impact numbers was lower in the fibrous mixtures than in the plain mixture, where the percentage increase in both L1 and L2 were lower for PF and SF mixtures than their corresponding percentages of the plain RC mixture. For instance, the incorporation of bedding materials increased L1 and L2 by 8 to 78% for RC specimens, while the percentages of increase for PF and SF specimens were in the ranges of 4% to 37%.
− Line-notched specimens and sand bedding significantly decreased COV of the testing results among the other suggested alterations. Using a cross-line notched specimen and line of impact with coarse bedding also effectively reduced COV for all mixtures. For example, SF-NN-NB, SF-ST-NB, SF-LN-NB, and SF-CN-NB specimens displayed a COV of approximately 23.2%, 20.9%, 7.4%, and 12.7% for L1 and 18.6%, 16.4%, 9.8%, and 13.9% for L2, respectively. Compared to the ACI testing method, all suggested test alterations exhibited a lower COV, which confirmed a controlled failure pattern.
− From the ACI testing method, a central semi-circular fraction zone was formed on the top surface of fibrous specimens. However, the lines and cross-notched specimens produced failures on two and four sides, respectively, distributing stress along a cross-line and controlling the failure path.

## Figures and Tables

**Table 1:** Summary of drop weight impact test results reported by earlier studies.

Refs.	Mixture ID	Quantity of Fiber	Type of Fiber	Number of Specimens Tested per Mix	Impact Numbers	Standard Deviation	Coefficient of Variation
[16]	PC, CF2, CF3, CF4, HF5, HF6, HF7	0.5, 1.0, 1.5	CSF, HSF	6	22, 26, 30, 36, 28, 32, 38	12, 11, 13, 17, 14, 15, 18	54, 44, 45, 47, 49, 46, 48
[17]	GHPPC, GHPFRC,	1.0	SF	16	33, 97	12, 30	-
[18]	PC, FRC1, FRC2, FRC3, FRC4, FRC5, FRC6	0.5, 1.0, 1.5	CSF, HSF	10	73, 164, 258, 338, 179, 269, 352	13, 19, 19, 20, 19, 22, 21	-
[19]	M0, M1, M2, M3	1.6, 0.3, 0.3	SF, PF, GF	5	14, 101, 32, 35	4.7, 20.3, 9.5, 11.7	33.5, 20.1, 30.1, 33.6
[20]	PC, CF1.5, CF3.0, CF5.0, HF1.5, HF3.0, HF5.0	1.5, 3.0, 5.0	CSF, HSF	15	84, 312, 737, 1209, 424, 918, 1378	25, 86, 113, 151, 64, 78, 122	30, 27, 15, 12, 15, 9, 9
[12]	PC, FRC, TSFRC, SIFCON	1.5, 4, 5, 8, 10	SF	6	36, 374, 1175, 1358, 1858, 2074	-	-
[21]	PC, LF1	2.5	SF	6	25, 232	10, 49	-
[22]	M1	2.5	SF	12	127	47	37
[23]	SC30-0, SC30-0.5, SC30-0.75, SC30-1.0	0.5, 0.75, 1.0%	SF	6	1.8, 7.3, 11.3, 17.2	0.8, 1.6, 1.6, 4.8	41.1, 22.3, 14.4, 27.9
[24]	CC, PAC1, PAC2	2.4%	SF, PF	15	16, 448, 110	7, 65, 26	-
[25]	ECC0-0, ECC2-0.5, ECC2-1, ECC2-1.5	0.5, 1.0, 1.5	PVA	3	1, 594, 697, 674	0, 4, 7, 6	-
[26]	NC, PP4, PP6, SF20, SF35	4, 6, 20, 35 kg/m^3^	PF, SF	6	15, 33, 40, 52, 55	7, 7, 5, 27, 24	47, 21, 12, 52, 44
[27]	GHPC, GHPSFRC	0.5	SF	40	177, 240	81, 94	46, 39
[28]	G1, G2	2.5%	SF	15	358, 417	207, 185	58, 44
[29]	B1, B2	3 kg/m^3^	PF	20	84, 76	44, 37	52, 49
[30]	PC, CFRC, PRFC, SFRC	0.15, 0.15, 0.5	CF, PF, SF	32	48, 118, 71, 228	28, 53, 36, 90	57, 45, 51, 39
[31]	HSFRC	1	HSF	48	1896	802	42
[32]	PFRC, FRC, SPHFRC	05, 1.5	PF, SF	-	52, 191, 267	26.7, 108.2, 89.6	51.2, 56.5, 33.5

Annotations: HSF; Hooked end steel fiber, CSF; Crimped steel fiber, PAC; Preplaced aggregate concrete, FRC; Fiber-reinforced concrete, PC; Plain concrete, TSFRC; Two stage FRC, HSFRC; High-strength fiber-reinforced concrete, SFRC; Steel FRC, PFRC; Polypropylene FRC, SIFCON; Slurry-infiltrated fibrous concrete, CFRC; Cellulose FRC, SF; Steel fiber, PF; Polypropylene fiber, CF; Cellulose fiber, GHPSFRC; green high-performance steel FRC, GHPC; green high-performance plain concrete, ECC; Engineered cementitious composite, PVA; polyvinyl alcohol.

**Table 2:** Mixing combination, notch, and bedding type.

Group	Mixture ID	Ratio of s/b	Ratio of w/b	Fiber Dosage (%)	Fiber Type	SP (%)	Notch Type	Bedding Type
1	RC-NN-NB	1.0	0.42	0	-	0.4	No notch	No bedding
	PF-NN-NB	1.0	0.42	2.4	PF	0.5		
	SF-NN-NB	1.0	0.42	2.4	SF	0.5		
2	RC-ST-NB	1.0	0.42	0	-	0.4		
	PF-ST-NB	1.0	0.42	2.4	PF	0.5		
	SF-ST-NB	1.0	0.42	2.4	SF	0.5		
3	RC-LN-NB	1.0	0.42	0	-	0.4	Line notch	
	PF-LN-NB	1.0	0.42	2.4	PF	0.5		
	SF-LN-NB	1.0	0.42	2.4	SF	0.5		
4	RC-CN-NB	1.0	0.42	0	-	0.4	Cross notch	
	PF-CN-NB	1.0	0.42	2.4	PF	0.5		
	SF-CN-NB	1.0	0.42	2.4	SF	0.5		
5	RC-NN-SB	1.0	0.42	0	-	0.4	No notch	Sand bedding
	PF-NN-SB	1.0	0.42	2.4	PF	0.5		
	SF-NN-SB	1.0	0.42	2.4	SF	0.5		
6	RC-ST-SB	1.0	0.42	0	-	0.4		
	PF-ST-SB	1.0	0.42	2.4	PF	0.5		
	SF-ST-SB	1.0	0.42	2.4	SF	0.5		
7	RC-LN-SB	1.0	0.42	0	-	0.4	Line notch	
	PF-LN-SB	1.0	0.42	2.4	PF	0.5		
	SF-LN-SB	1.0	0.42	2.4	SF	0.5		
8	RC-CN-SB	1.0	0.42	0	-	0.4	Cross notch	
	PF-CN-SB	1.0	0.42	2.4	PF	0.5		
	SF-CN-SB	1.0	0.42	2.4	SF	0.5		
9	RC-NN-CB	1.0	0.42	0	-	0.4	No notch	Coarse aggregate bedding
	PF-NN-CB	1.0	0.42	2.4	PF	0.5		
	SF-NN-CB	1.0	0.42	2.4	SF	0.5		
10	RC-ST-CB	1.0	0.42	0	-	0.4		
	PF-ST-CB	1.0	0.42	2.4	PF	0.5		
	SF-ST-CB	1.0	0.42	2.4	SF	0.5		
11	RC-LN-CB	1.0	0.42	0	-	0.4	Line notch	
	PF-LN-CB	1.0	0.42	2.4	PF	0.5		
	SF-LN-CB	1.0	0.42	2.4	SF	0.5		
12	RC-CN-CB	1.0	0.42	0	-	0.4	Cross notch	
	PF-CN-CB	1.0	0.42	2.4	PF	0.5		
	SF-CN-CB	1.0	0.42	2.4	SF	0.5		

**Table 3:** Impact test results.

Group	Mixture ID	Impact Numbers						Mean		SD		COV (%)	
		Specimen 1		Specimen 2		Specimen 3							
		L1	L2	L1	L2	L1	L2	L1	L2	L1	L2	L1	L2
1	RC-NN-NB	8	15	9	19	14	26	10	20	3.2	5.6	31.1	27.8
	PF-NN-NB	31	125	46	148	58	198	45	157	13.5	37.3	30.1	23.8
	SF-NN-NB	74	375	85	482	115	548	91	468	21.2	87.3	23.2	18.6
2	RC-ST-NB	10	21	14	25	18	31	14	26	4.0	5.0	28.6	19.6
	PF-ST-NB	40	138	49	164	60	203	50	168	10.0	32.7	20.2	19.4
	SF-ST-NB	81	389	93	492	121	542	98	474	20.5	78.0	20.9	16.4
3	RC-LN-NB	17	28	19	29	20	32	19	30	1.5	2.1	8.2	7.0
	PF-LN-NB	49	165	54	182	59	196	54	181	5.0	15.5	9.3	8.6
	SF-LN-NB	100	456	108	476	116	548	108	493	8.0	48.4	7.4	9.8
4	RC-CN-NB	18	31	21	36	25	39	21	35	3.5	4.0	16.5	11.4
	PF-CN-NB	52	174	59	195	68	222	60	197	8.0	24.1	13.4	12.2
	SF-CN-NB	99	426	115	525	128	561	114	504	14.5	69.9	12.7	13.9
5	RC-NN-SB	11	20	13	25	18	31	14	25	3.6	5.5	25.8	21.7
	PF-NN-SB	40	151	55	166	64	216	53	178	12.1	34.0	22.9	19.2
	SF-NN-SB	88	400	95	499	125	560	103	486	19.7	80.7	19.1	16.6
6	RC-ST-SB	15	26	18	30	21	35	18	30	3.0	4.5	16.7	14.9
	PF-ST-SB	54	163	65	180	72	217	64	187	9.1	27.6	14.3	14.8
	SF-ST-SB	89	432	100	489	119	563	103	495	15.2	65.7	14.8	13.3
7	RC-LN-SB	23	33	24	34	26	35	24	34	1.5	1.0	6.3	2.9
	PF-LN-SB	56	185	58	191	61	207	58	194	2.5	11.4	4.3	5.9
	SF-LN-SB	109	494	114	504	120	555	114	518	5.5	32.7	4.8	6.3
8	RC-CN-SB	25	35	26	39	31	41	27	38	3.2	3.1	11.8	8.0
	PF-CN-SB	56	196	61	216	69	241	62	218	6.6	22.5	10.6	10.4
	SF-CN-SB	106	505	121	555	128	612	118	557	11.2	53.5	9.5	9.6
9	RC-NN-CB	12	24	15	31	21	39	16	31	4.6	7.5	28.6	24.0
	PF-NN-CB	42	161	61	178	70	236	58	192	14.3	39.3	24.8	20.5
	SF-NN-CB	91	423	99	523	134	598	108	515	22.9	87.8	21.2	17.1
10	RC-ST-CB	16	32	20	39	24	44	20	38	4.0	6.0	20.0	15.7
	PF-ST-CB	58	176	67	197	79	237	68	203	10.5	31.0	15.5	15.2
	SF-ST-CB	91	473	105	501	126	615	107	530	17.6	75.2	16.4	14.2
11	RC-LN-CB	25	49	26	52	29	54	27	52	2.1	2.5	7.8	4.9
	PF-LN-CB	60	204	62	199	67	231	63	211	3.6	17.2	5.7	8.1
	SF-LN-CB	115	540	117	536	128	620	120	565	7.0	47.4	5.8	8.4
12	RC-CN-CB	28	48	31	51	36	59	32	53	4.0	5.7	12.8	10.8
	PF-CN-CB	59	205	64	225	74	256	66	229	7.6	25.7	11.6	11.2
	SF-CN-CB	109	530	129	561	135	646	124	579	13.6	60.1	10.9	10.4

## Data Availability

Data sharing not applicable.
